# Supplementary material for: The mediating role of knowledge sharing behavior and the moderating role of digital mindset: Evidence in Vietnam
Source: PLoS One. 2026 Mar 5;21(3):e0342798. doi: 10.1371/journal.pone.0342798 (PMC12962511; doi:10.1371/journal.pone.0342798)
Supplement: S1 Table — (DOCX) [file pone.0342798.s001.docx]

# **APPENDIX: Measurement Items**

| **No.** | **Measurement Items** | **Sources** |
| --- | --- | --- |
| **1** | **Reward system** |  |
| 1.1 | Sharing my knowledge will lead to recognition and appreciation. | Javaid et al. (2020) |
| 1.2 | Sharing my knowledge will lead to learning and training opportunities. |  |
| 1.3 | Sharing my knowledge will lead to additional credit toward promotion. |  |
| **2** | **Organizational commitment** |  |
| 2.1 | I would be glad to spend the rest of my career with this organization. | Luo et al. (2021); Sang et al. (2020) |
| 2.2 | This organization is personally very meaningful to me. |  |
| 2.3 | I was taught to value loyalty to an organization. |  |
| **3** | **Organizational strategy** |  |
| 3.1 | My firm has the capability to develop business strategies. | Wu & Chen (2014); Prieto & Revilla (2004) |
| 3.2 | My firm has the capability to implement both inter-firm and intra-firm collaboration. |  |
| 3.3 | My firm has the capability to ensure strategic alignment in information systems planning. |  |
| **4** | **Organizational structure** |  |
| 4.1 | Knowledge sharing practices increase when the organization supports informal meetings and exchanges. | Abdelwhab Ali et al. (2019) |
| 4.2 | Knowledge sharing practices increase when an open-space office environment is adopted. |  |
| 4.3 | Knowledge sharing practices increase when job rotation is implemented. |  |
| **5** | **Knowledge sharing** |  |
| 5.1 | Employees share business proposals and reports with each other. | Yang & Chen (2007) |
| 5.2 | Employees share success and failure stories with each other. |  |
| 5.3 | Employees share know-how and expertise gained from work experiences with each other. |  |
| **6** | **Knowledge application** |  |
| 6.1 | The organization is able to identify and apply knowledge to adapt to changing competitive conditions. | Ode & Ayavoo (2020); Turulja & Bajgorić (2020) |
| 6.2 | The organization has processes for using knowledge to solve new problems. |  |
| 6.3 | Our firm uses knowledge to improve efficiency. |  |
| **7** | **Digital mindset** |  |
| 7.1 | I am usually one of the first to try out new digital technologies. | Goldmann et al. (2025) |
| 7.2 | When encountering digital technologies, I assess their benefits and risks for my organization. |  |
| 7.3 | I view digital transformation as an opportunity rather than a threat. |  |
