# Supplementary material for: The mediating role of knowledge sharing behavior and the moderating role of digital mindset: Evidence in Vietnam
Source: PLoS One. 2026 Mar 5;21(3):e0342798. doi: 10.1371/journal.pone.0342798 (PMC12962511; doi:10.1371/journal.pone.0342798)
Supplement: Attachment — Submitted filename: Response to Reviewers.docx [file pone.0342798.s004.docx]

***Manuscript ID: PONE-D-25-42326***

***Title***: THE MEDIATING ROLE OF KNOWLEDGE SHARING BEHAVIOR AND THE MODERATING ROLE OF DIGITAL MINDSET: EVIDENCE IN VIETNAM

| **Editor comments** | **Authors’ explanations** |
| --- | --- |
| In the ethics statement in the Methods, you have specified that verbal consent was obtained. Please provide additional details regarding how this consent was documented and witnessed, and state whether this was approved by the IRB | The authors have revised the methodology section and adjusted based on the editor suggestions – **Page 13**.  The survey was conducted with experts, directors, and employees working at branches of commercial banks in Ho Chi Minh City, Vietnam, from February to June 2025. A total of 350 questionnaires were distributed via email and QR codes, and after excluding eight incomplete responses, 315 valid questionnaires were retained for the analysis, yielding a high response rate of 90%. Prior to accessing the questionnaire, participants were provided with an information sheet detailing the study’s objectives, procedures, potential risks and benefits, and data protection measures. Online informed consent was obtained by requiring participants to click “I agree to participate” before proceeding with the survey, and they were free to withdraw at any time. Only respondents who explicitly confirmed consent were able to complete the questionnaire. This procedure, including the use of online informed consent, was reviewed and approved by the Institutional Review Board/Ethics Committee of Saigon University, ensuring compliance with the Declaration of Helsinki. |
| Thank you for stating the following financial disclosure: “This research is funded by Saigon University” | “This research is funded by Saigon University” is stated at the end of the manuscript. |
| In the online submission form, you indicated that “The data supporting the findings of this study are available from the corresponding author, T.on request.” | The authors have provided the data and use **DRYAD to create its DOI.**  DOI: 10.5061/dryad.02v6wwqhh |
| Please include your full ethics statement in the ‘Methods’ section of your manuscript file. In your statement, please include the full name of the IRB or ethics committee who approved or waived your study, as well as whether or not you obtained informed written or verbal consent. If consent was waived for your study, please include this information in your statement as well. | The authors have revised the methodology section and adjusted based on the editor suggestions:  The survey was conducted with experts, directors, and employees working at branches of commercial banks in Ho Chi Minh City, Vietnam, from February to June 2025. A total of 350 questionnaires were distributed via email and QR codes, and after excluding eight incomplete responses, 315 valid questionnaires were retained for the analysis, yielding a high response rate of 90%. Prior to accessing the questionnaire, participants were provided with an information sheet detailing the study’s objectives, procedures, potential risks and benefits, and data protection measures. Online informed consent was obtained by requiring participants to click “I agree to participate” before proceeding with the survey, and they were free to withdraw at any time. Only respondents who explicitly confirmed consent were able to complete the questionnaire. This procedure, including the use of online informed consent, was reviewed and approved by the Institutional Review Board/Ethics Committee of Saigon University, ensuring compliance with the Declaration of Helsinki. |
| Please ensure that you refer to Figure 1 in your text as, if accepted, production will need this reference to link the reader to the figure. | The authors have included Figure 1 in the manuscript for reference – **Page 12**.  The research model (Figure 1) examines the impact of organizational factors, including reward system (RS), organizational commitment (OC), organizational strategy (OS), and organizational structure (OT), on knowledge sharing (KS). Furthermore, KS is expected to positively influence knowledge application (KA). In addition, digital mindset (DM) is hypothesized to play a moderating role, strengthening the effects of organizational factors on KS as well as the relationship between KS and knowledge application |
| Please include a copy of Table 6 which you refer to in your text on page 15. | The authors have corrected the wrong number of tables. For example: **Page 14**  “**Table 4**. R-square and R-square adjusted” is corrected to “**Table 3**. R-square and R-square adjusted” |
| Please include captions for your Supporting Information files at the end of your manuscript, and update any in-text citations to match accordingly. Please see our Supporting Information guidelines for more information:  <http://journals.plos.org/plosone/s/supporting-information>. | The authors have added Supporting Information files at the end of your manuscript:  **Supporting Information**  **S1 Table. Measurement model results.**  Composite reliability, Cronbach’s alpha, and AVE indices of all study constructs (Table 1).  **S2 Table. Discriminant validity results.**  HTMT ratio values confirming discriminant validity among constructs (Table 2).  **S3 Table. Coefficient of determination.**  R-square and adjusted R-square values of the dependent variables (Table 3).  **S4 Table. Collinearity and effect size assessment.**  VIF values and f-square indices for independent variables (Table 4).  **S5 Table. Hypothesis testing results.**  Path coefficients, t-statistics, and significance levels of the structural model (Table 5).  **S1 Figure. Research model.**  The conceptual model shows organizational factors, knowledge sharing, knowledge application, and digital mindset (Figure 1).  **S2 Figure. Structural model results.**  PLS-SEM bootstrapping results with standardized path coefficients (Figure 2).  **S1 Data. Survey dataset (anonymized).**  The dataset supporting the findings of this study, available from the corresponding author upon request |
| If the reviewer comments include a recommendation to cite specific previously published works, please review and evaluate these publications to determine whether they are relevant and should be cited. There is no requirement to cite these works unless the editor has indicated otherwise. | The authors have responded to the comments of reviewers. |
| Please review your reference list to ensure that it is complete and correct. If you have cited papers that have been retracted, please include the rationale for doing so in the manuscript text, or remove these references and replace them with relevant current references. Any changes to the reference list should be mentioned in the rebuttal letter that accompanies your revised manuscript. If you need to cite a retracted article, indicate the article’s retracted status in the References list and also include a citation and full reference for the retraction notice. | The authors have checked the References section and added the missing DOI. |
| **Additional Editor Comments:** |  |
| 1. Hypotheses Section:  Ensure that all hypotheses are correctly numbered and listed. Hypothesis 2 appears to be missing, and Hypothesis 4 is listed twice. | The authors have adjusted the numbering of the hypotheses accordingly. |
| 2. Consistent Use of Abbreviations:  Once introduced, abbreviations like KS (Knowledge Sharing) and AI (Artificial Intelligence) should be used consistently throughout the text to maintain clarity. | The authors have revised and consistently used the abbreviation 'KS (Knowledge Sharing)' throughout the manuscript. |
| 3. Headings and Section Introductions:  Several main and subheadings (e.g., 2., 2.1., 2.3.1.) begin abruptly. Please add short introductory sentences to guide the reader. | The authors have revised and added some transitional paragraphs as follows:   2.1. **Literature review - Page 4.**   This section provides an overview of the main theoretical foundations and previous studies that inform the research model. The discussion begins with key theories that explain knowledge sharing behavior, including Social Exchange Theory, the Theory of Planned Behavior, and Social Cognitive Theory. It then incorporates Dynamic Capabilities Theory and Sociotechnical Systems Theory to highlight the role of digital mindset in knowledge management and application….  **4.1. Research results – Page 13**  This section presents the empirical results obtained from the PLS-SEM analysis. The findings include assessments of reliability and validity, discriminant validity, explanatory power of the model, effect sizes, and hypothesis testing, supported by tables and figures.  **4.2. Discussions – Page 16**  This section discusses the key findings of the study in relation to existing theories and prior empirical research. The analysis highlights how organizational factors and digital mindset influence knowledge sharing and application, and it evaluates the consistency of the results with previous studies. |
| 4. Literature Review Flow:  Consider merging or streamlining subsections to reduce fragmentation and improve readability. | The authors would like to sincerely thank the reviewer for the valuable comments. Following this suggestion, we have revised the manuscript by grouping the related theories into two main categories: (i) Theories Explaining Individual Behavior and (ii) Theories of Dynamic Capabilities and Socio-Technical Systems. This approach makes the theoretical framework clearer, more coherent, and better aligned with the research objectives.  This section provides an overview of the main theoretical foundations and previous studies that inform the research model. The discussion begins with key theories that explain knowledge sharing behavior, including Social Exchange Theory, the Theory of Planned Behavior, and Social Cognitive Theory. It then incorporates Dynamic Capabilities Theory and Sociotechnical Systems Theory to highlight the role of digital mindset in knowledge management and application.  **2.1.1. Theories explaining individual behavior**  Social Exchange Theory (SET) suggests that social relationships are formed and maintained on the basis of exchanging benefits, as indicated by Homans (1958); Blau (1964). Accordingly, social behavior only occurs when the benefits received are considered greater than the costs incurred. In organizational settings, SET is often used to explain the motivation for knowledge sharing, which is considered a trade-off between costs and benefits (Cropanzano & Mitchell, 2005). When the work environment creates favorable conditions such as trust among colleagues, support from superiors, or a fair organizational culture, employees tend to actively share knowledge – a voluntary behavior that depends on subjective perceptions of exchange value (Cook et al., 2013; Lin, 2007).  SET emphasizes external interactions and social situations that facilitate knowledge sharing and exchange. At the same time, Social Cognitive Theory (SCT) provides an additional explanation in terms of internal psychological mechanisms. According to this theory, human behavior is the result of interactions among personal factors (beliefs and skills), behavior, and environment (Bandura (1982). Regarding knowledge-sharing behavior, SCT emphasizes the importance of self-efficacy in promoting proactive behavior. It means that employees who believe they are capable of sharing knowledge effectively will tend to be more proactive in communicating information, supporting colleagues, and promoting innovation at the organizational level (Hsu et al., 2007; Safdar et al., 2021).  Besides SET and SCT, the Theory of Planned Behavior (TPB) emphasizes the role of behavioral intention in forming knowledge-sharing behavior. According to Ajzen (1991), attitudes, subjective norms, and perceived behavioral control are key factors that determine an individual's intention, which in turn influences their behavior. In the organizational context, organizational commitment reflects a positive attitude toward knowledge sharing, while perceived competence in knowledge sharing reflects perceived behavioral control (Ajzen, 1991; Fishbein & Ajzen, 2011). Combining the three theories helps to explain better how knowledge sharing behavior stems from personal motivation and beliefs, and is influenced by the social environment, thereby promoting innovation in organizations.  **2.1.2. Theories of Dynamic Capabilities and Socio-Technical Systems**  In the era of substantial digital transformation, digital mindset – the way each individual shapes their perception and readiness to embrace technology – is increasingly seen as a decisive factor, helping organizations convert knowledge into practical innovation activities (Kane et al., 2015; Warner & Wäger, 2019). This mindset enables individuals to adapt swiftly to technological changes, leverage digital tools, and creatively apply knowledge to drive innovation.  According to the Dynamic Capabilities Theory of Teece et al. (1997), dynamic capabilities are understood as the ability of a business or individual to integrate, build, and restructure internal and external resources, including knowledge, to adapt to an ever-changing environment. In this framework, digital thinking is viewed as a manifestation of dynamic capabilities at the individual level, demonstrating the ability to flexibly adapt to new technologies and quickly exploit and apply knowledge in a digital context (Warner & Wäger, 2019).  Furthermore, according to Socio-Technical Systems Theory, an organization consists of the social part (people and structure) and the technical part (technology and processes). When these two parts work together, knowledge sharing and innovation will occur more effectively (Mumford, 2006; Trist & Bamforth, 1951). In this context, a digital mindset refers to one’s ability to identify, understand, and apply competently within a technology system to increase the effectiveness of KS and its application. People who have developed a digital mindset are more skilled in learning, technology use, and work practice innovation; therefore, they are more likely to adapt with less difficulty, especially in more automated and digitalized organizational contexts (Kane et al., 2015). |
| 5. Results Section:  + Improve clarity of table descriptions. + Ensure statistical values such as β coefficients are introduced properly, not just during hypothesis confirmation. + Explain the meaning of key findings more directly, rather than reiterating theory. | The authors have revised Results section based on the reviewers’ comments.  First, Digital Mindset (DM) has a statistically significant positive effect on KA because the coefficient is 0.228 415 and its p-value is 0.004 000 (less than 5%)… The research results indicate that businesses need to focus on cultivating digital thinking for both leaders and employees to enhance technology adaptability, promote innovation and improve competitiveness. From a theoretical perspective, this finding highlights the role of digital thinking as a distinct form of dynamic capability during the digital transformation period and confirms that the connection between people and technology is the key factor in effectively exploiting and applying knowledge.  Second, the impact of KS on KA is strong and statistically significant (β = 0.511, p = 0.000). This confirms that KS is essential for bridging knowledge ownership and application actions at work… This result confirms that KS has a positive impact on KA, meaning that the more effectively knowledge is shared, the easier it is for employees to transform knowledge into concrete actions. Knowledge sharing not only helps spread experience and skills within the organization but also creates a collaborative learning environment, encourages innovation, and supports faster problem-solving. Therefore, promoting knowledge sharing is a crucial strategy for improving the quality of knowledge application and enhancing the competitiveness of enterprises.  Organizational Commitment (OC) significantly affects KS (β = 0.353167, p = 0.001000)… The findings suggest that high levels of employee commitment to the organization encourage knowledge sharing as a way of expressing commitment and gratitude. This commitment creates a positive cycle: more committed employees share more, and sharing in turn improves performance and strengthens their trust in the organization. Therefore, creating a fair work environment, recognizing contributions, and providing career development opportunities are key to enhancing organizational commitment and promoting knowledge sharing behavior.  Moreover, Organizational Strategy (OS) also positively impacts KS (β = 0.142100, p = 0.010007), suggesting that strategic orientations that emphasize innovation, learning, and internal collaboration will create a favorable foundation for KS… From the results, it is suggested that organizational strategy plays a significant role in shaping knowledge sharing behavior, as shared development goals encourage employees to learn from and share experiences with one another. A strategy designed to support knowledge will help break down departmental barriers, create a collaborative culture, and improve innovation performance. Therefore, embedding KS into long-term vision and development strategy is an important factor in maintaining sustainable competitive advantage.  In addition, organizational structure (OT) positively affects KS (β = 0.296, p = 0.000),… A flat and open organizational structure facilitates peer-to-peer collaboration, reduces administrative barriers, and promotes rapid and widespread knowledge sharing. Designing a flexible organization that promotes horizontal connections, and open communication is crucial to enhancing the effectiveness of knowledge sharing within an organization. |
| 6. Discussion on Sustainability:  As suggested by Reviewer 2, consider incorporating a brief section connecting your findings to ESG (Environmental, Social, Governance) goals to enhance contextual depth. | The authors have incorporated an additional paragraph that discusses the study’s implications for sustainability.  **5.1. Conclusions**  In addition to the theoretical and managerial contributions, the research results also have important implications for the implementation of ESG (Environmental, Social, Governance) goals. First, knowledge sharing and organizational commitment contribute to strengthening the social dimension through increased engagement, cooperation, and collective learning. Organizational strategies oriented towards innovation and knowledge sharing are closely linked to the Environmental dimension when promoting sustainable practices and efficient use of resources. In addition, digital thinking and fair reward mechanisms clearly reflect the Governance dimension, emphasizing transparency, accountability, and responsible technology management. Therefore, developing digital thinking and encouraging knowledge sharing within corporations enhances their innovation capacity and competitive advantage, while also supporting the achievement of ESG goals and contributing to long-term sustainable development. |
| 7. Data Availability:  PLOS ONE requires that data be publicly available unless legal or ethical restrictions apply. Please update your Data Availability Statement with a justification or ensure the data is deposited in a public repository. | The authors have uploaded data based on the requirement of PlosOne. |
