# Supplementary material for: The mediating role of knowledge sharing behavior and the moderating role of digital mindset: Evidence in Vietnam
Source: PLoS One. 2026 Mar 5;21(3):e0342798. doi: 10.1371/journal.pone.0342798 (PMC12962511; doi:10.1371/journal.pone.0342798)
Supplement: Attachment — Submitted filename: Response to Reviewers_round 3.docx [file pone.0342798.s006.docx]

***Manuscript ID: PONE-D-25-42326***

***Title***: THE MEDIATING ROLE OF KNOWLEDGE SHARING BEHAVIOR AND THE MODERATING ROLE OF DIGITAL MINDSET: EVIDENCE IN VIETNAM

| **Reviewer 1’s comments** | **Authors’ explanations** |
| --- | --- |
| All comments have been addressed | Thank you so much Reviewer 1 for the careful reading and constructive feedback. We sincerely appreciate your valuable comments, which have helped us improve the clarity, rigor, and overall quality of the manuscript. We have addressed all of Reviewer 1’s comments point-by-point in the revised version and believe the paper is now substantially strengthened. |

| **Reviewer 3’s comments** | **Authors’ explanations** |
| --- | --- |
| All comments have been addressed | Thank you so much Reviewer 3 for the careful reading and constructive feedback. We sincerely appreciate your valuable comments, which have helped us improve the clarity, rigor, and overall quality of the manuscript. We have addressed all of Reviewer 1’s comments point-by-point in the revised version and believe the paper is now substantially strengthened. |
