# Supplementary material for: The mediating role of knowledge sharing behavior and the moderating role of digital mindset: Evidence in Vietnam
Source: PLoS One. 2026 Mar 5;21(3):e0342798. doi: 10.1371/journal.pone.0342798 (PMC12962511; doi:10.1371/journal.pone.0342798)
Supplement: Attachment — Submitted filename: Reviewer Comment Plos One.docx [file pone.0342798.s005.docx]

Reviewer Comment:

The paper is empirically solid, with robust use of a staggered Difference-in-Differences (DID) model and various checks, but it has room for enhancement to strengthen its academic rigor, clarity, and impact. The core explanatory variable "Policy" is a dummy (1 if a city opens facilities in a given year, 0 otherwise), which overlooks nuances like the number, type (e.g., wastewater vs. air quality facilities), scale, or frequency of public access. This binary approach may oversimplify the policy's intensity and heterogeneous effects, potentially leading to measurement error or attenuated results. The limitations section acknowledges this but does not explore it deeply.

Under developed Literature Review and Novelty Framing The introduction highlights contributions (e.g., multi-agent regulation, targeted greenwashing measure), but the literature review is brief and China-centric. It cites relevant studies but does not deeply contrast with international contexts (e.g., EU or US disclosure regulations) or emerging theories like stakeholder salience or signalling theory in greenwashing.

Reliance on Specific Data Sources with Potential Coverage Gaps Greenwashing is measured as the absolute difference between standardized Bloomberg ESG disclosure scores and CNRDS ESG performance scores. While innovative, these databases may have incomplete coverage for smaller or non-heavy-polluting Chinese firms, leading to selection bias in the sample (reduced from 21,000 to 4,416 observations). The sample period (2011–2023) is post-policy heavy, but pre-policy trends might be under-represented.

Limited Exploration of Endogeneity Beyond Standard Checks The paper uses PSM-DID, parallel trends, placebo tests, and fixed effects effectively, but does not employ instrumental variables (IV) or other advanced methods to address potential reverse causality (e.g., firms influencing policy implementation) or omitted variables (e.g., local political cycles).

Narrow Scope of Heterogeneity and Mechanisms The motivation (government/public pressure, costs) and heterogeneity (green investors, analysts, subsidies) analyses are strong but limited. For example, it does not explore firm-level traits like ownership type (state-owned vs. private) or industry pollution intensity, which could moderate effects.

Dense Writing and Long Sentences Some sections (e.g., introduction, hypotheses) have lengthy sentences that reduce readability (e.g., multi-clause explanations of mechanisms). Tables are clear, but figures (e.g., parallel trends) lack detailed captions explaining confidence intervals.

Incomplete Limitations and Future Research The limitations section covers data binary nature, unexamined moderators, and archival method limits well, but it is brief and does not quantify impacts (e.g., how binary measure might bias coefficients downward).

Overall, this is a well-executed article with strong empirical contributions to greenwashing literature. Addressing these revisions could elevate it for top journals like PLOS ONE. Prioritize methodological refinements and clarity for the next revision.
